# Variability of Breast Surface Positioning Using an Active Breathing Coordinator for a Deep Inspiration Breath Hold Technique

**DOI:** 10.7759/cureus.15649

**Published:** 2021-06-14

**Authors:** Kristen McConnell, Neil Kirby, Karl Rasmussen, Alonso N Gutierrez, Nikos Papanikolaou, Dennis Stanley

**Affiliations:** 1 Department of Radiation Oncology/Medical Physics, University of Alabama at Birmingham, Birmingham, USA; 2 Department of Radiation Oncology, University of Texas Health Science Center at San Antonio, San Antonio, USA; 3 Department of Radiation Oncology, Miami Cancer Institute, Miami, USA; 4 Department of Radiation Oncology, University of Alabama at Birmingham, Birmingham, USA

**Keywords:** dibh, surface imaging, catalysthd, elekta abc, medical physics

## Abstract

Purpose

The Elekta Active Breathing Coordinator^TM^ (ABC) is used to control breathing and guide deep inspiration breath hold (DIBH). It has been shown to be accurate in lung cancers, but limited analysis has been performed on the spatial accuracy and reproducibility of the breast surface. The use of optical surface-image guidance for patient positioning has grown in popularity and is an alternative solution for breast DIBH. This study aims to evaluate the breast surface variability of an ABC-guided DIBH by using a three-dimensional (3D) surface imaging system to record surface position.

Methods

Ten participants were placed in the treatment position, and breathing baselines and inhalation volume threshold baselines were monitored and recorded using the ABC. Over 60 minutes, the breathing patterns were recorded by the ABC and CatalystHD^TM^ (C-RAD, Uppsala, Sweden). For each breath hold, the valve of the ABC closed at the baseline inhalation threshold and a 3D surface image was acquired. For each point on the baseline breast surface, a 3D vector was calculated to the subsequent breath hold surface as well as a root mean square (RMS) vector magnitude for the entire surface.

Results

The average and standard deviation for the RMS difference between the baseline and subsequent evaluated images were 7.12 ± 2.70 mm.

Conclusion

This study shows that while the ABC-guided inhalation volume is kept constant, a non-negligible variability of the breast surface position exists. Special considerations should be used in clinical situations, where the positioning of the surface is considered more important than inhalation volume.

## Introduction

When delivering radiotherapy to the left breast, increased cardiac morbidity and mortality are of concern [1]. Deep inspiration breath hold (DIBH) is a technique that has been shown to decrease the mean heart dose while maintaining coverage of the breast [2-8]. In the DIBH technique, a patient performs a breath hold to a specified level during each delivery of radiation. A common choice for controlled DIBH delivery involves using an active breathing coordinator (ABC) system (Elekta, Stockholm, Sweden) to provide information on a patient’s predicted location of anatomy. The ABC utilizes a spirometer-based valve system to control breathing to serve as a guide for DIBH.

The underlying assumption is that a breath hold reaching the same volume indicates consistent internal anatomical positioning. The use of DIBH coupled with the ABC for lung cancer treatment has been shown to reduce critical organ dose [9-11], but there have been studies indicating that lung volume is not a perfect proxy for anatomical position. Plathow et al. showed that lung and chest wall position was dependent on the type of breathing maneuver [12]. The study used dynamic magnetic resonance imaging (MRI) and a fiducial marker to measure the differences in position of the lung and chest wall during three breathing maneuvers: “abdominal breathing”, “thoracic breathing”, and “normal breathing”. This could indicate that using the ABC for DIBH may result in positioning errors due to breathing maneuvers.

Recently, the use of optical surface image guidance has grown in popularity and is an alternative solution for accurate breast DIBH tracking [13,14]. With this method, the tracking does not rely on the inspiration volume but rather on the motion of the patient surface. The underlying assumption for this technique, as with all surface imaging systems, is that anatomical location is consistent with the surface location.

Coupling the use of an ABC to fix the inhalation volume with a surface image of each breath hold has the potential to identify any positioning errors that could result from different breathing maneuvers that manifest during a typical treatment. An abstract published by Flampouri et al. in 2008 used a surface imager coupled with an ABC to compare and measure inter-breath hold and intra-breath hold [15]. They reported a 2.0-mm surface difference for the inter-breath hold (between two successive breaths) and a 2.5-mm surface difference for the intra-breath hold (movement within each breath). This study utilized an AlignRT (VisionRT, London, Great Britain) surface imaging system. In our study, 10 participants held their breath to a fixed volume as measured by the ABC. A C-RAD CatalystHD^TM^ three-dimensional (3D) surface imaging system (C-RAD, Uppsala, Sweden) was used to acquire a surface image during the breath hold, which was subsequently compared to the reference breath hold to measure differences in position.

The abstract of this article was presented at the American Association of Physicists in Medicine (AAPM) Annual Meeting, July 30 to August 3, 2017, Denver, CO.

## Materials and methods

Elekta Active Breathing Coordinator^TM^

The ABC is a spirometer-based breathing control system consisting of a flexible, disposable mouthpiece connected to a breathing tube, a turbine cartridge to measure volume flow, a balloon valve to control volume flow, a nose clip for limiting air through the nostrils, and a control system [9]. Breathing patterns, both baseline inhalation and breath hold volumes, were established adhering to the recommended standard clinical setup with the ABC in place.

C-RAD CatalystHD

**Figure 1:**
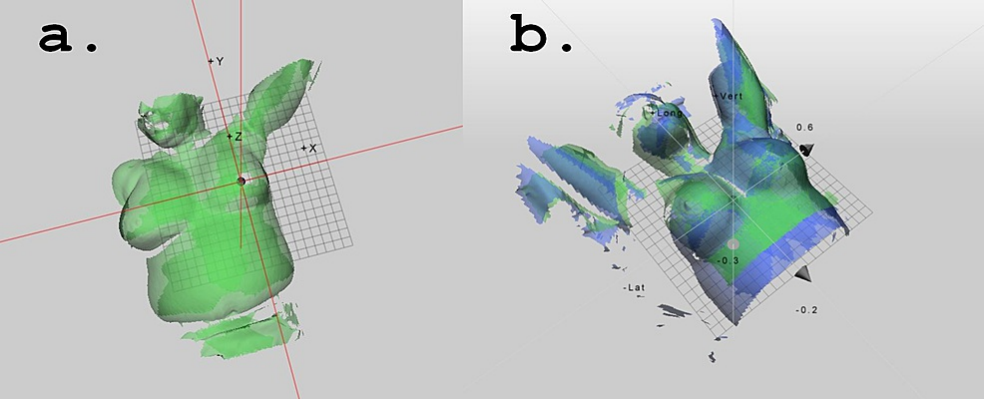
(a) Representative reference image (green) and (b) the real-time patient position (blue) superimposed onto the reference image (green) with calculated shifts for each direction.

The C-RAD CatalystHD is a ceiling-mounted three-camera optical-based imaging system capable of monitoring patient setup and positioning, detecting intra-fraction and inter-fraction motion, and performing respiratory gating. The cameras are mounted at fixed angles so that localization and visualization of the patient are maintained for every gantry location by projecting specific light patterns of a known wavelength (405 nm: near-invisible violet; 528 nm: green; and 624 nm: red) onto the patients’ skin. The CatalystHD algorithm then matches the light pattern that is projected onto the patients’ surface to a reference surface acquired previously (either from the reference CT or from baseline surface scan performed prior to treatment). The CatalystHD algorithm registers the two patterns and provides the patient positioning errors, expressed in six degrees of freedom, required to put the patient into the correct position. Figure 1a shows a representative reference image (green) and Figure 1b shows the real-time patient position (blue) superimposed onto the reference image (green) with calculated shifts for each direction.

Study design

**Figure 2:**
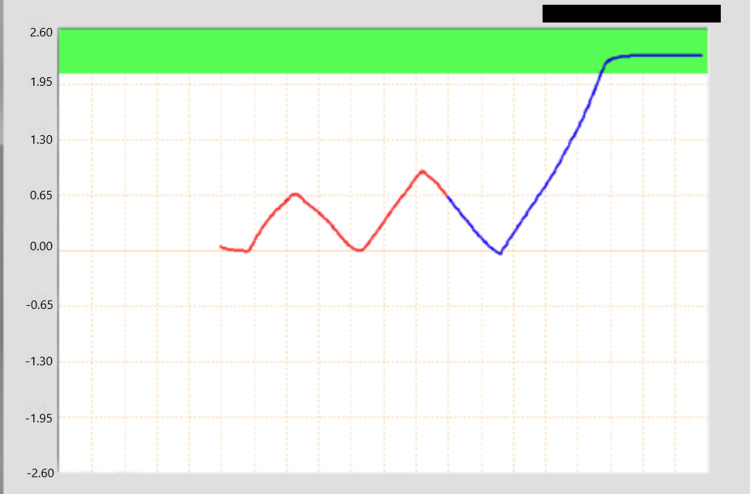
The ABC screen shows a multicolored line, which represents the ventilation signal of the participant. The red portion indicates a free breathing state while the blue indicates that the participant has enabled the ABC to trigger when the correct inspiration threshold (green band) is achieved. ABC, Active Breathing Coordinator

In this study, the ABC was used to monitor the inspiration volume for 10 female participants (age = 35.1 ± 12.7 years), while the CatalystHD was used to monitor surface positioning for comparison. To begin, the participants were positioned in a standard clinical left breast DIBH setup using a breast board, set at a standard angle of 10 degrees, with the ABC system in place. A free-breathing pattern was recorded using the ABC, and then participants were asked to hold their breath to obtain the maximum inspiration volume. Next, a moderate DIBH (mDIBH), set at 80% of the subject-specific maximum inspiration volume, was established. Figure 2 shows the mDIBH threshold for a representative patient. This value was used as the trigger for closing the valve of the ABC; once a participant reached that level, the valve would close, signaling the participant to hold their breath.

For each participant, during the first breath hold, a reference CatalystHD image was acquired. This reference image served as the baseline with which all subsequent breath hold evaluation images could be compared. Throughout the entirety of the study, a fixed, patient-specific inhalation volume was achieved using the ABC. Simultaneously, the CatalystHD was used to acquire an evaluation 3D surface image. This process was repeated over the course of 60 minutes, providing 30 CatalystHD 3D surface images associated with each subsequent ABC breath hold. Additionally, as per IRB regulations, the participants of this study were all briefed on the operation of both the ABC and the CatalystHD and were all familiar with the clinical workflows.

Analysis

**Figure 3:**
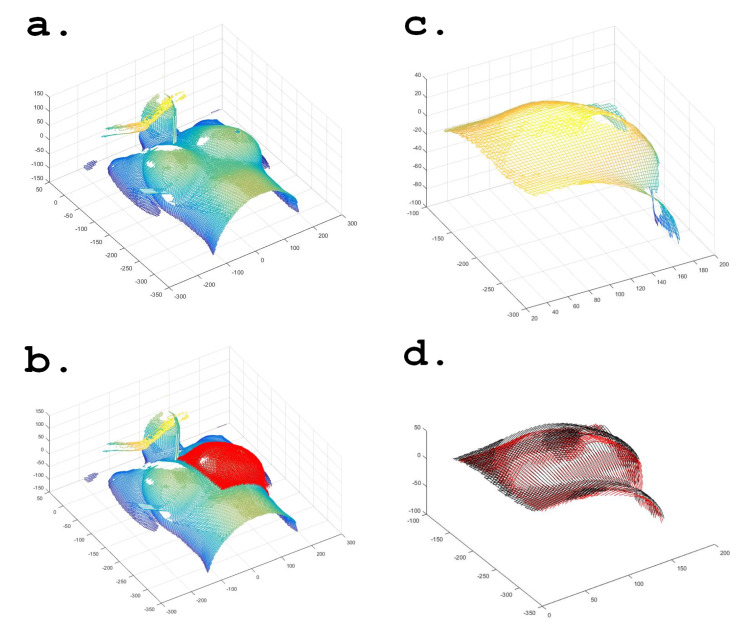
(a) Reference breath hold image as a 3D surface, (b) user-defined breast region overlaid on the reference image, (c) representative evaluation image of selected breast region, and (d) reference (red) and evaluation (black) images overlaid.

The raw surface images were imported into MATLAB® (MathWorks, Natick, MA), reconstructed as 3D surfaces, and the left breast region of the image was manually contoured on each image and used for analysis. Figure 3a shows the reference breath hold image acquired at the beginning of the study reconstructed as a 3D surface. Figure 3b shows the user-defined breast region of the evaluation image, outlined in red, overlaid on the reference 3D surface image. The selection of this region was made to be representative of the entire clinical breast volume. Figure 3c shows a representative evaluation image with the breast region selected. Figure 3d shows the reference (red) and the evaluation (black) images charted on top of each other. MATLAB was used to calculate the root mean square (RMS) vector error between the reference and evaluation images. For each point on the reference image, a 3D vector was calculated by finding the nearest point on the reference image that matched the closest point on the evaluation breath hold surface. These 3D vectors were used to find an RMS vector error across the entire 3D surface to produce an overall 3D positional error.

## Results

Table 1 shows a summary of the average and standard deviation of the 3D RMS difference and normalized inhalation threshold volume. Special care and consideration were taken to ensure that no systematic errors obscured the results. The final 3D error vectors were analyzed to check for any time dependency in an effort to realize any pattern of errors that may arise from patient relaxation during the study. Due to the limited sample size and study regulations designated by the IRB, further investigation of the dependency on breast size is needed.

**Table 1:** Summary of the average and standard deviation of the 3D RMS difference and normalized inhalation threshold volume. RMS, root mean square

3D RMS Statistical Values	
Average difference (mm)	7.12 ± 2.70
Maximum difference (mm)	11.72
Minimum difference (mm)	1.02
Median difference (mm)	7.67
Normalized inhalation threshold volume (L/L)	1.0 ± 0.0

## Discussion

As the technology of radiation therapy and delivery continues to improve, the need for effective and efficient motion management has become paramount. Many common treatment sites, such as the breast, lung, and liver, are prone to large target movements and uncertainties as a result of standard respiratory motion. Prior to the availability of motion management techniques, an expansion of the treatment volume was commonly used to account for the uncertainties. As the popularity and availability of motion management systems have increased, management protocols, such as DIBH, have become integral to the reduction of critical organ dose and motion assessment/reduction. The use of the ABC during DIBH techniques has been shown to reduce dose to critical organs [8-11] and is commonly used to control DIBH techniques. Kaza et al. showed that lung volumes controlled with an ABC were better than when self-controlled during the same session [16]. Consequently, the ABC can provide reproducible lung volumes, but the underlying assumption for some sites, specifically the breast, is that consistent lung volumes provide consistent positioning. The difference in positioning based on breathing technique suggests that even with a fixed inhalation volume, if a patient changes breathing type, the positioning could change as well.

Alternatively, the use of surface imaging for DIBH has grown recently and provides many advantages including decreased setup time, increased setup accuracy [17], real-time motion management, and no ionizing radiation. Alderliesten et al. showed that using 3D surface imaging for DIBH in patients with left-sided breast cancer was an accurate motion management technique [13]. While surface imaging systems have an inherent deficiency in the ability to visualize internal anatomy, the prevailing benefit is the ability to monitor both the respiratory signal and patient position simultaneously. Using the surface of the patient to monitor, the respiratory signal eliminates the concern that changes in inhalation volume or breathing type could produce a measurable difference in the positioning of the target volume. Additionally, as volumetric modulated arc therapy with a simultaneous integrated boost (VMAT-SIB) for the treatment of breast cancer becomes more prevalent [18-20], the importance of correct positioning of the entire breast surface becomes imperative to accurate dose deposition.

It is important to consider the relevance of motion metrics to the tumor location: volume of air in the lungs for the ABC or the position of the breast surface in space for the CatalystHD. Our results showed that even though the inspiration volume, as measured by the ABC, was held constant, the images captured by the CatalystHD indicated that, on average, a 7.12 ± 2.70 mm difference between the reference and evaluation images existed. Several studies have shown that differences on this magnitude could have potential effects on the toxicity to vital organs at risk, such as the heart and lungs [21-24]. This suggests that the ABC alone cannot accurately guarantee correct positioning of the breast during DIBH.

## Conclusions

This study showed that even though inhalation volume was kept constant, a non-negligible variability of the breast surface position exists. Special considerations should be used in clinical situations, such as the treatment of breast cancer, where the positioning of the surface is considered more important than inhalation volume.
